# Neurogenic to Gliogenic Fate Transition Perturbed by Loss of HMGB2

**DOI:** 10.3389/fnmol.2017.00153

**Published:** 2017-05-23

**Authors:** Robert Bronstein, Jackson Kyle, Ariel B. Abraham, Stella E. Tsirka

**Affiliations:** ^1^Program in Neuroscience, Stony Brook University, Stony BrookNY, United States; ^2^Cold Spring Harbor Laboratory, Cold Spring HarborNY, United States; ^3^Molecular and Cellular Pharmacology Graduate Program, Department of Pharmacological Sciences, Stony Brook University, Stony BrookNY, United States

**Keywords:** HMGB2, histone modification, postnatal neurogenesis, epigenetics, chromatin

## Abstract

Mouse cortical development relies heavily on a delicate balance between neurogenesis and gliogenesis. The lateral ventricular zone produces different classes of excitatory pyramidal cells until just before birth, when the production of astroglia begins to prevail. Epigenetic control of this fate shift is of critical importance and chromatin regulatory elements driving neuronal or astroglial development play an vital role. Different classes of chromatin binding proteins orchestrate the transcriptional repression of neuronal-specific genes, while allowing for the activation of astrocyte-specific genes. Through proteomic analysis of embryonic neural progenitor cells (NPCs) our group had previously identified high mobility group B2 (HMGB2), a chromatin protein dynamically expressed throughout embryonic development. In the current study using cultures of perinatal NPCs from HMGB2^+/+^ and HMGB2^-/-^ mice we discovered that vital elements of the polycomb group (PcG) epigenetic complexes polycomb repressive complexes 1 and 2 (PRC1/2) were downregulated during the differentiation process of HMGB2-null NPCs. These epigenetic changes led to downstream changes in specific histone modification levels, specifically the trimethylation of H3K27, and a subsequent shift in the perinatal neurogenesis to gliogenesis fate transition. Collectively these results demonstrate that chromatin binding proteins, such as HMGB2, can have significant effects on the epigenetic landscape of perinatal neural stem/progenitor cells.

## Introduction

Neural stem/progenitor cells (NSPCs) persist in discrete areas of the postnatal mouse brain (Abraham et al., 2013a). In the ventricular zone (VZ) and sub-ventricular zone (SVZ) of the lateral ventricles, NSPCs proceed through sustained waves of neurogenesis (∼E10–E17.5) and gliogenesis (∼E17.5–p10). These peaks of differentiation are not mutually exclusive: a small glial component is produced in the embryonic period, and a small neuronal component is generated in the early postnatal period. There is, however, a delicate and time precise balance that has to be maintained—from the proper neuronal lamination of the cerebral cortex to the functional integration of astrocytes and myelinating oligodendrocytes into the parenchymal tissue (Abraham et al., 2013b).

Cortical neurogenesis (∼E10–E17.5) proceeds through a highly stereotyped cascade, whereby radial glial fibers serve as a migratory scaffold for the inside out lamination of the cortical plate (Cao et al., 2014). These bipolar progenitors extend their arbors to make contact with the glia limitans dorsally, and the VZ ventrally (Abraham et al., 2013a). Radial glial endfeet therefore have extensive access to signals from the meninges, as well as the cerebrospinal fluid in the embryonic lateral ventricles. As neurogenesis begins to wane toward the end of the embryonic period, this pool of VZ progenitors undergoes a genetic fate switch—giving rise to three primary brain-derived glial subtypes: (1) astrocytes, (2) oligodendrocytes, (3) NG2^+^ cells (Corley and Kroll, 2015). Postnatally, NSPCs retract their apical fibers from the pial surface, and become restricted to VZ/SVZ, where they are supported by a vast plexus of blood vessels, all the while maintaining their ventricular contacts (Decoville et al., 2001).

Specific genetic programs serve to underpin cell fate transitions in the brain, such as the perinatal shift from cortical neurogenesis to gliogenesis. Polycomb group (PcG) and trithorax group (TrxG) proteins are well known epigenetic mediators and play key roles in chromatin dynamics during this period (Déjardin et al., 2005). First characterized in the Drosophila embryo, they are critical in patterning the body plan, as they bind to polycomb responsive elements (PREs) to mediate their modulatory effects (Egan et al., 2013). Many proteins are associated with and contribute to PcG-modulated transcriptional regulation, and they are subdivided into two complexes in the mouse, polycomb repressive complex 1 (PRC1) and polycomb repressive complex 2 (PRC2). The mammalian PRC2 includes three core proteins critical for gene repression: enhancer of zeste 2 (EZH2), embryonic ectoderm development (EED), and suppressor of zeste 12 (SUZ12) (Gabellini et al., 2002)—a complex primarily involved in catalyzing the mono-, di-, and tri-methylation of histone H3K27 (Gruber et al., 2011; Gonzales-Roybal and Lim, 2013). Combinatorial activity of PcG and TrxG in stem and progenitor cells regulates cell-type-specific fate acquisition and differentiation reversibly through the co-modification of gene promoters with both the “active” H3K4me3 and “repressive” H3K27me3 modifications (He et al., 2005).

After a shotgun proteomic screen of proliferating and differentiating mouse embryonic NSPCs, we reported that the chromatin architectural protein high mobility group B2 (HMGB2) was expressed with a high degree of temporal variability—leading us to further probe its role in NSPC dynamics (Hirabayashi et al., 2009; Hirabayashi and Gotoh, 2010). Dorsal switch protein 1 (DSP1), the Drosophila homolog of HMGB2, has been classified as an enhancer of trithorax and polycomb (a group of proteins modulating the activity of PcG and TrxG) (Huang, 2014), and shown to form multimeric complexes—serving as a prominent recruiter of polycomb repressive factors to specific PREs during switches in cell fate (Hwang et al., 2014). Knockdown of EED, a critical component of PRC2, has been demonstrated to result in a delayed neurogenic to gliogenic fate shift in E11.5 NSPCs (Corley and Kroll, 2015). Given the epigenetic role played by DSP1, we reasoned that the absence of expression of HMGB2 in NSPCs may affect the time course and chromatin characteristics of differentiated progeny (Jobe et al., 2012; Kim and Kim, 2012; Hwang et al., 2014). We sought to assess the functional contribution of murine HMGB2 in the regulation of PcG proteins and consequent downstream effects during the perinatal transition from cortical neurogenesis to gliogenesis.

## Materials and Methods

### Animals and Genotyping

All animal experiments performed have been approved by and are in agreement with the guidelines of the Stony Brook University (SBU) Institutional Animal Care and Use Committee (IACUC). HMGB2 null breeding pairs on a C57BL/6 background were obtained from Dr. Lorenza Ronfani (Core Facility for Conditional Mutagenesis, San Raffaele Scientific Institute, Milan, Italy). The HMGB2 deficient mouse line was created by conventional gene targeting methods (Kokovay et al., 2010). Due to the sterility of the HMGB2 homozygous null males (Kokovay et al., 2010), two breeding strategies were employed for the following experiments: heterozygous females were crossed with heterozygous males or homozygous females crossed with heterozygous males. In instances where Mendelian ratios did not yield adequate numbers of HMGB2^+/+^ controls (e.g., the second breeding scheme), age-matched controls were substituted from non-transgenic C57BL/6 breeding. The HMGB2^-/-^ animals are smaller in size, but do not initially appear grossly phenotypically different than wild-type animals.

### Primary Adherent Monolayer Culture of NSPCs

Pups ranging in age from postnatal day 0 to postnatal day 2 were used for all cell culture experiments. Brains were extracted and placed in sterile Hank’s buffered salt solution (HBSS) containing 2% glucose. From each hemisphere—olfactory bulbs, hippocampi, midbrain, cerebellum, and meninges were dissected away and discarded. The remaining brain regions, VZ, SVZ, striatum, and minimal underlying cortex, were placed in 5 mL of HBSS supplemented with 2% glucose. The tissue was triturated in 1 mL of complete Neurocult neural progenitor cells (NPC) Basal Proliferation Media with the addition of 20 ng/mL of recombinant human epidermal growth factor (rh EGF), 10 ng/mL recombinant human basic fibroblast growth factor (rh bFGF), 0.2% heparin, and 5% penicillin/streptomycin. The tissue suspension was spun down at 1500 rpm at room temperature. The subsequent tissue pellet was resuspended in 200 μl of complete Neurocult NPC Basal Proliferation Media with the addition of 20 ng/mL of rh EGF, 10 ng/mL rh bFGF, 0.2% heparin, and 5% penicillin/streptomycin. The tissue was further triturated with a pipette tip until a uniform cell suspension was achieved. The cell suspension was filtered through a 40 μm nylon mesh cell strainer and plated on six-well plates precoated with 100 μg/mL poly-D-lysine (PDL, 1 h), and 10 μg/mL laminin (4 h). Following approximately 2–3 days of proliferative growth, the cells were replated in precoated 60 mm tissue culture plates in complete Neurocult differentiation media containing supplements and 5% penicillin/streptomycin.

### Histone Extraction and Immunoblotting

Histones were obtained from cell culture lysates at various time points primarily employing the method described in Kriegstein and Alvarez-Buylla (2009). Briefly, cells were centrifuged at room temperature at 300 *g* and the pellets cleaned with 10 mL of sterile HBSS. The pellets were resuspended in 1 mL of hypotonic lysis buffer (10 mM Tris–Cl pH 8.0, 1 mM KCl, 1.5 mM MgCl2, 1 mM dithiothreitol (DTT), protease and phosphatase inhibitors; Kriegstein and Alvarez-Buylla, 2009) and rotated at 4°C for 30 min. Following this step, a nuclear pellet was obtained by spinning the hypotonically dissociated cells at 4°C, 10,000 *g* for 10 min. Following this step the NSPC nuclear pellet was resuspended in 400 μl of H_2_SO_4_ and rotated overnight at 4°C. Nuclear debris was removed by spinning the acid extracted preparation at 16,000 *g* for 10 min. A total of 132 μl of trichloroacetic acid was added to the supernatant to a 33% final concentration to precipitate the histones. A final spin step was used to pellet the purified histones and two subsequent washes with ice-cold acetone cleared away any remaining acid. The histones were dried for 20 min at room temperature and resuspended in 100 μl of ddH_2_O. Concentrations of purified histone proteins were obtained via ImageJ densitometry measurements following 15% SDS-PAGE and subsequent coomassie staining. Immunoblots utilized the aforementioned percentage gels followed by transfer to polyvinylidene difluoride membranes. The following antibodies were used to probe for specific histone modifications: (1) Tri-Methyl-Histone H3 (Lys27), Rabbit monoclonal antibody (mAb) from Cell Signaling (#9733) at a concentration of 1:1000, (2) Di/Tri-Methyl-Histone H3 (Lys9), Mouse IgG1 mAb from Cell Signaling (#5327) at a concentration of 1:2000, (3) Tri-Methyl-Histone H3 (Lys4), Rabbit mAb from Cell Signaling (#9751) at a concentration of 1:1000, (4) Ubiquityl-Histone H2A (Lys119) Rabbit mAb from Cell Signaling (#8240) at a concentration of 1:2000, (5) Anti-Histone H3 Rabbit polyclonal from Abcam (ab1791) at a concentration of 1:2500, (6) Anti-Histone H2A Rabbit polyclonal from GeneTex (GTX129418).

### Quantitative Real-Time PCR Arrays

The following Qiagen RT^2^ Profiler 84-gene arrays were utilized: Mouse Polycomb & Trithorax Complexes (PAMM-506Z). The arrays were loaded with cDNA reverse transcribed from RNA which was obtained from 24-h differentiation cultures of HMGB2^+/+^ and HMGB2^-/-^ NSPCs. An Applied Biosystems StepOnePlus Quantitative Real-Time PCR (qRT-PCR) machine served as the PCR platform. Data were analyzed initially on the Qiagen web-based analysis software followed by Microsoft Excel (Supplementary Figure 1).

### Quantitative Real-Time PCR

Total RNA was obtained from differentiating HMGB2^+/+^ and HMGB2^-/-^ NSPCs at days 1, 3, and 5. RNAbee (Amsbio) was used for RNA extraction followed by purification via the Qiagen RNeasy Mini Kit (74104). Subsequently cDNA was reverse transcribed using the High-Capacity cDNA Reverse Transcription Kit (Life Technologies—4368814). GAPDH was used as housekeeping gene. For gene expression analysis, the relative quantitation method was used (ΔΔCt) with GAPDH as an internal control. All primer sequences are listed in Supplementary Table 1.

### Immunocytochemistry/Immunofluorescence

HMGB2^+/+^ and HMGB2^-/-^ NSPCs were differentiated for 3 or 7 days in eight-well chamber slides (Fisher-Mediatech 177445) precoated with 100 μg/mL PDL (1 h), and 10 μg/mL laminin (4 h) in a tissue culture incubator at 37°C. HMGB2^+/+^ and HMGB2^-/-^ NSPCs were differentiated for 3 or 7 days in eight-well chamber slides (Fisher-Mediatech 177445) precoated with 100 μg/mL PDL (1 h), and 10 μg/mL laminin (4 h) in a tissue culture incubator at 37°C. Individual chambers were blocked for 1 h with phosphate buffered saline (PBS) containing 5% normal goat serum, 1% bovine serum albumin (BSA), and 0.3% Triton-X100. Antibody solutions were made up of PBS containing 1% BSA, and 0.03% Triton-X100. Immunocytochemistry/immunofluorescence antibodies are listed in Supplementary Table 2.

P1 wild-type (WT) and HMGB2 knock-out (KO) brains were collected and passively fixed in 4% paraformaldehyde (PFA), whereas p21 WT and KO brains were perfused with 4% PFA and postfixed with 4% PFA for 12 h. After fixation, brains were put in 30% sucrose until they were no longer floating, and then frozen in OCT via dry ice. Cryostat sectioning at 20 μm thickness was done, and slides were stored in -80°C.

For staining, slides were allowed to get to room temperature, and then washed in PBS. Blocking was done for 2 h at room temperature with 5% serum and 0.3% Triton X-100. Primary antibodies were diluted with 0.01% BSA and 0.3% Triton X-100, and incubated at 4°C overnight or 2 h at room temperature. Slides were washed in PBS, and then incubated with Alexa fluorophore secondary antibodies at 1:1000 dilution for 1 h at room temperature. After another PBS wash, slides were cover slipped with 4′,6-diamidino-2-phenylindole (DAPI) Fluoromount.

Imaging was done on a SP8X Leica Confocal Microscope. Images shown are 20 μm z-stacks.

### Statistics

For comparisons between groups of two an unpaired, two-tailed *t*-test was employed. For multiple comparisons within a group a one-way ANOVA was used followed by either Holm–Sidak multiple comparison *post hoc* analysis, or Bonferroni’s *post hoc* multiple comparisons test. GraphPad Prism and Microsoft Excel were used for all statistical analyses. A 95% confidence interval is used for all figures indicating initial significance at *p* < 0.05 marked by single asterisk “^∗^” and greater significance at *p* < 0.01 marked by double asterisks “^∗∗^”. Standard error of the mean (SEM) is the calculation underlying all graph error bars, *n* refers to the number of biological replicates used in each experiment.

## Results

### Polycomb Complex Genes Downregulated in Differentiating NSPCs from HMGB2^-/-^ Mice

**FIGURE 1:**
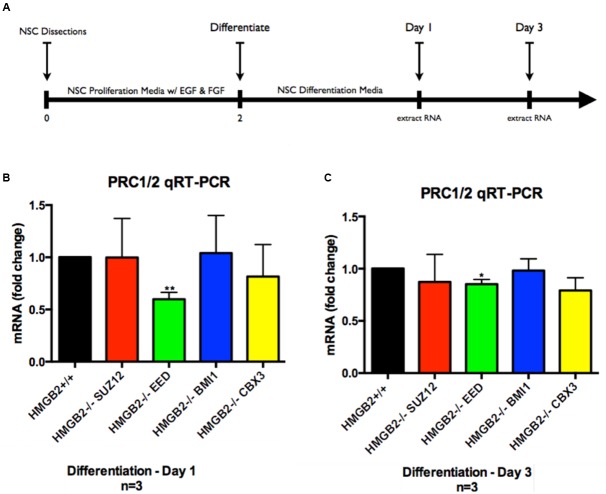
**Assessment of genes downregulated in PcG and TrxG arrays via qRT-PCR.** Following differentiation of NPCs for 1 and 3 days cDNA was obtained for verification of gene expression patterns obtained via qRT-PCR arrays. Representative experimental timeline demonstrating 2 days of primary NPC proliferation followed by differentiation of 1 and 3 days. **(A)** Time course of cell culture experiment. **(B)** qRT-PCR results from samples obtained at day 1 of differentiation showing fold-change differences for four key PcG subunit complex genes, with EED showing significant downregulation in the HMGB2^-/-^ samples as compared with HMGB2^+/+^. **(C)** qRT-PCR results from samples obtained at day 3 of differentiation showing fold-change differences for four key PcG subunit complex genes, with EED showing significant downregulation in the HMGB2^-/-^ samples as compared with HMGB2^+/+^. *n* = 3 per genotype. ^∗∗^EED at day 1 *p* = 0.0038; ^∗^EED at day 3 *p* = 0.0109. To calculate fold change values the HMGB2^+/+^ levels for each gene were set to 1 (black bar).

Monolayer NSPC cultures from HMGB2^+/+^ and HMGB2^-/-^ p0–p2 pups (isolated from primary dissections of SVZ) were grown in media promoting cellular proliferation (bFGF and rh EGF) until the plates were ∼85% confluent. The cells were replated in 60 mm dishes containing differentiation media. Following 24 h of differentiation the cells were harvested and total RNA was extracted for 84-gene analysis via the Qiagen qRT-PCR array system—specifically looking at genes encoding key PcG and TrxG subunits (Supplementary Figure 1). In the PcG and TrxG arrays key PRC1 and PRC2 subunits (EED, SUZ12, CBX3, BMI1) were revealed to be downregulated in the HMGB2^-/-^ NSPCs following 1 day of differentiation (Supplementary Figure 1). Although many other complex macromolecular assemblies have built-in redundancies making the elimination of one component tolerable, these key PcG genes encode critical pieces of PRC1 and PRC2 complexes (Gonzales-Roybal and Lim, 2013). To further refine the analysis following the initially shallow Qiagen screen, we sought to verify which of these components was downregulated at days 1 and 3 of NSPC differentiation using qRT-PCR. Primary NSPCs were plated; after 2 days of proliferative growth they were exposed to differentiation conditions through the removal of growth factors from the media. RNA was extracted at 1 and 3 days following the induction of differentiation (**Figure 1**). Our findings indicate that EED, an important member of PRC1 and PRC2 is significantly downregulated by 50% in HMGB2^-/-^ NPCs at day 1 of differentiation (**Figure 1A**), and by 20% at day 3 of differentiation (**Figure 1B**). The expression of the remainder of the genes examined, SUZ12, BMI1, and CBX3, was not significantly modified (indicating that while the array produced important leads, it would only be an initial step in the analysis). Lower EED expression was evident in p1 HMGB2^-/-^ SVZ tissue compared to the expression in HMGB2^+/+^ animals, although the difference was not statistically significant, possibly reflecting EED protein stability (Supplementary Figure 2).

Collectively these results point to a critical role for the chromatin protein HMGB2 in the dynamic regulation of EED, a key polycomb complex component, and potentially the expression of cell fate genes regulated by PcG.

### Repressive Epigenetic Histone Modifications in Differentiating HMGB2-Null NSPCs

**FIGURE 2:**
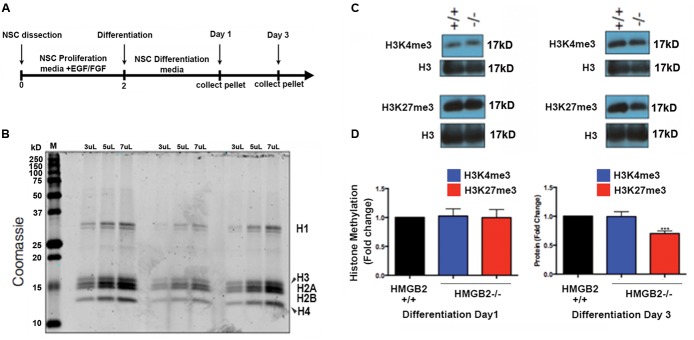
**Changes in repressive histone modifications in differentiating NSPCs.** **(A)** Time course and outline of the experimental procedure. **(B)** Histone proteins were purified with sulfuric acid (H_2_SO_4_) and precipitated using trichloroacetic acid; to quantify protein amounts proteins were electrophoresed on a polyacrylamide gel and visualized with coomassie. M, signifies the molecular weight ladder. **(C)** Immunoblots employing antibodies against the histone modifications H3K4me3 and H3K27me3, which are permissive and repressive, respectively. Total histone H3 represents the loading control. **(D)** Quantification of the histone methylation western blot results showing reduction in H3K27me3 at 3 days of NPC differentiation in the HMGB2^-/-^ NSPCs. *n* = 3–4 biological replicates per genotype. ^∗∗∗^H3K27me3 from HMGB2^-/-^ extracts at day 3 *p* = 0.0001. To calculate fold change values the HMGB2^+/+^ levels for each modified histone protein over the relevant H3 levels were set to 1.

EED is a core component of the PRC2 complex catalyzing the methylation of histone H3 at lysine 27 (K27; Lamiable et al., 2010). The presence of tri-methylation on H3K27 can function as signal to recruit PRC1 complexes to H3K27me3 marks, and thus initiate the process of gene silencing and chromatin condensation. EED also acts to stabilize H2A ubiquitin E3 ligase activity (Gruber et al., 2011). It is therefore responsible for the coordination of PRC1 and PRC2 activities, which are critical to the repression of specific neuronal and astroglial genes (Gruber et al., 2011; Lim and Alvarez-Buylla, 2014) and the onset of stem cell differentiation (Lamiable et al., 2010). To examine whether global levels of H3K27me3 were altered in HMGB2^-/-^ NSPCs we utilized the monolayer NSPC culture system from which we acid-extracted histone proteins at days 1 and 3 of differentiation (**Figures 2A,B**). H3K27me3 levels were visualized using immunoblots; they remained the same 1 day following the initiation of differentiation in the HMGB2^+/+^ and HMGB2^-/-^ NSPCs, however, they were significantly reduced at day 3 of differentiation in the HMGB2^-/-^ cultures (**Figures 2C,D**).

We also evaluated another repressive histone modification, H3K9me3, which is laid down by a different epigenetic complex that does not include EED (Margueron and Reinberg, 2011). No changes were observed between HMGB2^+/+^ and HMGB2^-/-^ NPCs at differentiation (Supplementary Figure 3), suggesting HMGB2 may be specifically involved in the regulation of EED function. The mechanism regulating this putative negative feedback loop remains to be elucidated.

### Gliogenesis Is Perturbed in Differentiating NSPCs from HMGB2^-/-^ Mice

**FIGURE 3:**
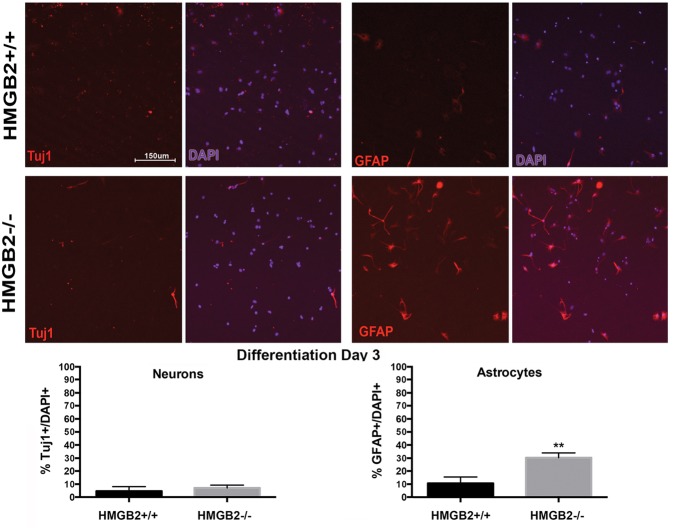
**Ratios of neural and glial cells altered in HMGB2^-/-^ SVZ cultures at day 3 of differentiation.** NSPCs were cultured in eight-well chamber slides with growth factor-free media and allowed to differentiate. Chamber slides from HMGB2^+/+^ and HMGB2^-/-^ NPCs stained for the immature neuronal marker Tuj1, all results represented as percentage of Tuj1^+^ (red) cells to all 4′,6-diamidino-2-phenylindole^+^ (DAPI^+^) (blue)nuclei. Chamber slides from HMGB2^+/+^ and HMGB2^-/-^ NPCs stained for the astrocyte marker GFAP, all results represented as percentage of GFAP^+^ (red) cells to all DAPI^+^ (blue) nuclei. *n* = 3 biological replicates per genotype. Sixty cells were counted per biological replicate. ^∗∗^GFAP *p* = 0.0049. Scale bar = 150 μm.

**FIGURE 4:**
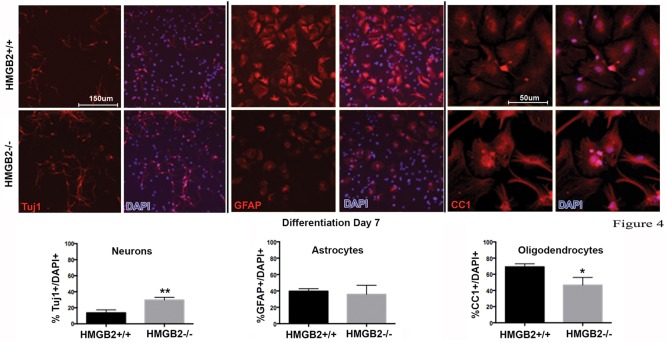
**Ratios of neural and glial cell altered in HMGB2^-/-^ SVZ cultures at day 7 of differentiation.** NPCs were cultured in eight-well chamber slides with growth factor-free media and allowed to differentiate. Chamber slides from HMGB2^+/+^ and HMGB2^-/-^ NPCs stained for the immature neuronal marker Tuj1, all results represented as percentage of Tuj1^+^ (red) cells to all DAPI^+^ (blue) nuclei. Chamber slides from HMGB2^+/+^ and HMGB2^-/-^ NPCs stained for the astrocyte marker GFAP, all results represented as percentage of GFAP^+^ (red) cells to all DAPI^+^ (blue) nuclei. *n* = 3 biological replicates per genotype, 60 cells were counted per biological replicate. Chamber slides from HMGB2^+/+^ and HMGB2^-/-^ NPCs stained for the oligodendrocyte marker CC1, all results represented as percentage of CC1^+^ (red) cells to all DAPI^+^ (blue) nuclei. *n* = 3 biological replicates per genotype. ^∗∗^Tuj1 *p* = 0.0054, ^∗^CC1 *p* = 0.0189. Scale bar = 150 μm, except for the CC1 images where the scale bar indicates 50 μm.

To assess the differentiation properties of perinatal NSPCs monolayer cultures were induced to differentiate in eight-well chamber slides for 3 days and 7 days, followed by immunostaining with cell-type specific antibodies. At day 3 of differentiation significantly more GFAP^+^ astrocytes were present in the HMGB2^-/-^ wells compared to WT, while neuronal populations, characterized by Tuj1^+^ staining, remained at near undetectable levels (**Figure 3**). Oligodendrocyte differentiation was not assessed at this timepoint, as the differentiation process to mature oligodendrocytes is not complete, and not all NG2^+^ cells become oligodendrocytes. Interestingly at day 7 of differentiation the percentage of Tuj1^+^ cells increased in the HMGB2^-/-^ wells (**Figure 4**). At this time point there was no difference in the numbers of GFAP^+^ astrocytes between the two genotypes; immunostaining for CC1 revealed a decrease in oligodendrocytes in the HMGB2^-/-^ wells. These data point to a possible role of HMGB2 loss on the neurogenic to gliogenic fate transition that occurs in perinatal NSPCs (**Figure 4**; Corley and Kroll, 2015). In addition, our previous experimental findings in the adult HMGB2^-/-^ mouse brain corroborate these perturbed perinatal cell fate transitions with a greater proportion of adult newborn olfactory bulb interneurons present in the adult HMGB2^-/-^ animal (Hirabayashi et al., 2009). This also is supported by the observation that there are apparent structural differences in CNS anatomy in the HMGB2^-/-^ mice. Fifty percent of the animals (*n* = 14) displayed enlarged ventricles at 10 weeks of age, while only 1 of 10 of HMGB2^+/+^ mice exhibited any detectable ventricular enlargement at that age (Supplementary Figure 4). Therefore, the effects of HMGB2 depletion may be additive throughout development and into the adult mouse brain.

**FIGURE 5:**
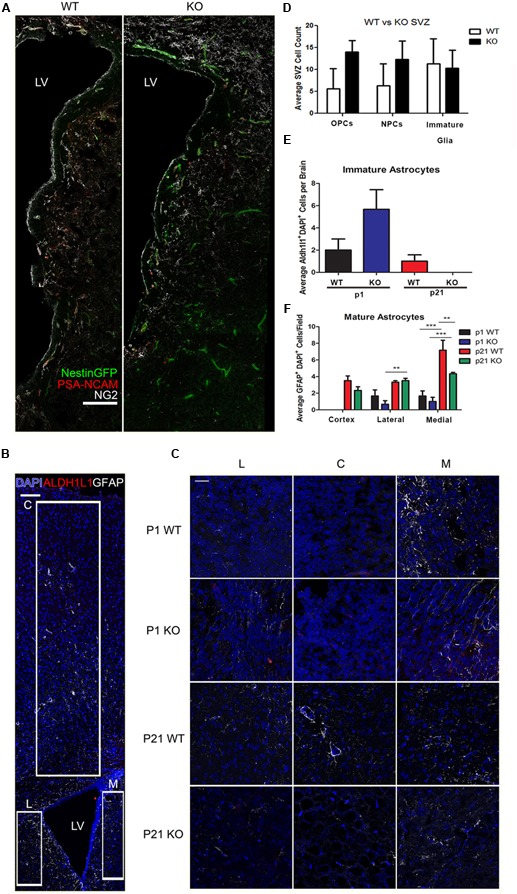
**Effect of HMGB2 KO on brain cell populations *in vivo*. (A)** Representative images of p1 HMGB2^+/+^ (WT) and HMGB2^-/-^ (KO) SVZ, with NestinGFP (green), PSA-NCAM (red), and NG2 (white). Scale bar = 100 μm. **(B)** Representative image of a p21 lateral ventricle, with DAPI (blue), the immature astrocyte marker Aldh1l1 (red), and the mature astrocyte marker GFAP (white). Boxes outline areas where 63× images were taken blindly for quantification of astrocytes for the lateral striatum, cortex, and medial striatum. Scale bar = 100 μm. **(C)** Representative images of the lateral striatum (L), cortex (C), and medial striatum (M), with DAPI (blue), Aldh1l1 (red), and GFAP (white). Scale bar = 25 μm. **(D)** Quantification of oligodendrocyte precursor cells (OPCs), NPCs, and immature glial cells in p1 WT and KO brains. OPCs were defined as PSA-NCAM^+^/NG2^+^, NPCs as PSA-NCAM^+^/NG2^-^, and immature glial cells as NG2^+^/PSA-NCAM^-^. *p* = 0.0508 for OPCs, *p* = 0.1868 for NPCs, Student’s *t*-test. **(E,F)** Depict two 63× images taken for lateral striatum, cortex, and medial striatum, for a total of six images per brain. **(E)** Quantification of Aldh1l1^+^ immature astrocytes. Aldh1l1^+^ cells from the different sections were added together and averaged across brains. *p* = 0.1570 for p1. Student’s *t*-test. **(F)** Quantification of GFAP^+^ mature astrocytes. ^∗^*p* < 0.05, ^∗∗^*p* < 0.01, ^∗∗∗^*p* < 0.001, two-way ANOVA. *n* = 3 for all groups.

To further assess the differentiation properties of neural stem cells (NSCs) lacking HMGB2, p1 and p21 HMGB2^+/+^ (WT) and HMGB2^-/-^ (KO) brains were sectioned and imaged (**Figures 5A–C**). As mentioned earlier, neurogenesis switches to give rise to glial cells (Corley and Kroll, 2015). Glial (PSA-NCAM^+^/NG2^+^) and neuronal (PSA-NCAM^+^/NG2^-^) progenitors were counted along the SVZ, with p1 HMGB2 KO mice having increased numbers of progenitors (**Figure 5D**). There was no change in the immature glial cell population (NG2^+^/PSA-NCAM^-^). Additionally, astrocytes were further examined. Immature (Aldh1l1^+^) and mature (GFAP^+^) astrocytes were evaluated in both the striatum and cortex (**Figures 5B,C**). A trend toward an increase of immature astrocytes in p1 KO was evident compared to WT, but by p21 there was no change between WT and KO mice (**Figure 5E**). There was no difference in the mature astrocyte population at p1, as GFAP expression is low and mature astrocytes are just now starting to form by p1 (**Figure 5F**; Mohn et al., 2008; Ming and Song, 2011). However, by p21, there was a decrease in the numbers of mature astrocytes in the medial striatum of KO mice compared to WT mice (**Figure 5F**). These data further support a role of HMGB2 in regulating the neurogenic/gliogenic fate, and support a prolonged neurogenic phase.

## Discussion

In the experiments described in this study we identified a potential role for HMGB2 in the epigenetic dynamics of NSPC chromatin. We report that the germline loss of the HMGB2 gene and the resulting chromatin architectural protein that it encodes contribute to a significant reduction in the expression of the vital PcG gene EED, with concomitant reduction in total H3K27me3 levels in NSPC histone lysates. These specific genetic changes in the HMGB2^-/-^ NSPC population may be linked with the altered fate transitions observed in p0–p2 mouse NSPCs when the proliferating cells are allowed to differentiate into the three principal CNS cell types: neurons, astrocytes, and oligodendrocytes (Obier et al., 2015). We do not think that the phenotypes observed in the HMGB2^-/-^ cells in our study could be due to the fact that the HMGB2 ablation is global and throughout development, as the cells do appear wild-type-like in many of the parameters assessed. One parameter examined was potential compensation by the other members of the HMGB family. Immunohistochemical evaluation of HMGB1 revealed a trend toward a modest upregulation of this protein in the HMGB2^-/-^ animals (Supplementary Figure 5), which was not statistically significant. However, in support of our findings, especially on the trimethylation levels of K27 on H3, a recent report by Monte et al. (2016) described a similar decrease in H3K27me3 when HMGB2 was knocked down in neonatal rat ventricular myocytes using HMGB2 siRNA.

In the Drosophila model system DSP1/HMGB2 is critical for recruitment of PcG complexes to specific PRE sites on chromatin. DSP1 binding to PREs induces the recruitment of PcG proteins and PcG-mediated silencing of certain active loci, such as those responsible for the control of variegation. While HMGB2 may also have a similar role to play in mammalian systems, we report a novel negative feedback loop involved with the control of PcG subunit gene expression (Hwang et al., 2014). We and others have reported that HMGB2 interacts with Oct4 dimers (Hirabayashi et al., 2009; Kriegstein and Alvarez-Buylla, 2009). It has been speculated that instead of only mediating PcG-repression, Hmgb2 may serve to maintain a permissive chromatin environment at loci where Oct4 is bound. The process may involve Akt activation thus linking it to cell cycle progression, DNA damage repair, and the regulation of cell fate transitions (Kriegstein and Alvarez-Buylla, 2009). Although the analysis of chromatin modifications included all gene loci (total lysates), EED has been shown to interact with the promoters of specific pro-neuronal and pro-glial genes, inhibiting the expression of their gene products at specific developmental time-points (Ronfani et al., 2001; Shechter et al., 2007).

Epigenetic modifiers can have profound effects on the fate transitions of cells including NSPCs in the perinatal SVZ (Shechter et al., 2007; Sparmann et al., 2013; Lim and Alvarez-Buylla, 2014; Corley and Kroll, 2015). Germline deletion of HMGB2 effects on the neurogenesis/gliogenesis fate transitions were assessed in a cell culture NSPC monolayer model, along with which molecular players potentially drive those differences. We identified that the key PcG gene EED is significantly downregulated during NSPC differentiation, with a subsequent reduction in the repressive histone mark H3K27me3, in agreement with a recent report in a different system. EED is critically important for catalyzing this trimethylation reaction. The changes were accompanied by greater numbers of neuronal cells present in the HMGB2^-/-^ cultures at day 7 of differentiation as compared with HMGB^+/+^. Reduction of EED mRNA levels, and thereby the accompanying PcG core subunit it encodes, has been associated with prolonged neurogenesis and delayed gliogenesis (Shechter et al., 2007; Abraham et al., 2013b; Steelman et al., 2016). Our results support this correlation as we observed a plateau in the numbers of cells differentiating toward astrocytes at day 7, and a significant reduction in differentiation toward oligodendrocytes. It is conceivable that the changes observed in the levels of histone marks specifically catalyzed by the PRC1/2 complexes come as a result of HMGB2 absence in the NSPCs, thereby limiting the expression levels of core polycomb complex genes.

Aberrant elongation of the neurogenic phase in mouse cortical development can have many negative consequences, including the improper lamination of cerebral cortex as well as aberrant micro/macro circuit development. Such changes can be clearly observed in various animal models of human congenital microcephaly, such as the MCPH1-del mouse line—made to recapitulate the neurodevelopmental disorder known as primary microcephaly 1 (Steffen and Ringrose, 2014). Similarly, a large increase in adulthood-evident hydrocephalus/ventriculomegaly in the HMGB2^-/-^ mouse brain may be indicative of mosaicism in cortical development due to the germline loss of this critical chromatin structural regulator. Our data suggest that the elimination of HMGB2 in the perinatal NSPC cultures alters the developmental timing—whereby the beginning of the gliogenic phase may be shifted to later time points in the HMGB2^-/-^ cells allowing neurogenesis to persist even at later stages. With gliogenesis starting later in the life of the animal, critical events such as astrocytic integration into cortical circuits as well as oligodendrocyte-mediated myelination of white matter tracts, such as the corpus callosum, are delayed—resulting in stunted cortical development overall. In fact in animals at 10 weeks of age we found that GFAP staining of NestinGFP^+^HMGB2^-/-^ brain sections without ventriculomegaly revealed greater GFAP staining in the SVZ compared to age-matched WT mice, although the degree of this increase in GFAP staining in these HMGB2^-/-^ mice without ventriculomegaly was not significant (data not shown). Therefore it seems reasonable to consider that HMGB2 absence in NSPCs, and the subsequent downregulation of EED gene expression can have similar downstream effects on perinatal cell fate choice. A number of other epigenetic factors could also be contributors to this overall process, such as DNMT complexes and TrxG elements, which together constitute major drivers of mammalian neurogenesis (Testa, 2011).

## Author Contributions

RB and ST designed the study. RB, JK, and AA carried out the experiments. RB, JK, and ST wrote the manuscript and designed the figures. RB, ST, JK, and AA edited the manuscript.

## Conflict of Interest Statement

The authors declare that the research was conducted in the absence of any commercial or financial relationships that could be construed as a potential conflict of interest.
