# Supplementary material for: Neurogenic to Gliogenic Fate Transition Perturbed by Loss of HMGB2
Source: Front Mol Neurosci. 2017 May 23;10:153. doi: 10.3389/fnmol.2017.00153 (PMC5440561; doi:10.3389/fnmol.2017.00153)
Supplement: Supplementary file 1 [file Data_Sheet_1.docx]

Supplementary Material

**Neurogenic to Gliogenic Fate Transition Perturbed by Loss of HMGB2**

Robert Bronstein^1^, Jackson Kyle^2^, Ariel B. Abraham^2^ and Stella E. Tsirka^1,2*^

^1^Program in Neuroscience, ^2^Molecular and Cellular Pharmacology Graduate Program and Department of Pharmacological Sciences, Stony Brook University, Stony Brook, NY 11794 USA

Corresponding Author: Stella E Tsirka, Pharmacological Sciences, BST8-192, Stony Brook University, Stony Brook NY 11794-8651. Email: [styliani-anna.tsirka@stonybrook.edu](mailto:styliani-anna.tsirka@stonybrook.edu)

**Supplementary Figure 1: PcG and trxG complex array targets identifies genes involved in neurogenesis downregulated in HMGB2-/- SVZ cultures as compared with WT. A.** Heat map and software validation of the targets are shown. HMGB2^+/+^ and HMGB2^-/-^ cDNA samples from 24 hours of differentiation are loaded into the 84-gene Qiagen qRT-PCR PcG and TrxG complex array. The experiment was performed as per manufacturer’s instructions, and the results were analyzed based on the process and software provided by Qiagen. To ascertain that only the most highly different genes were evaluated, we set an arbitrary 6-fold expression difference cutoff, and appraised a subset of genes that met these criteria. Each box contains the gene symbol along with software validation as to the likelihood that the qRT-PCR reaction is reliable, with OKAY indicating statistically reliable CT values across samples. N=1 **B.** The scatter plot compares gene expression levels between the two NSC genotypes. It plots the log10 of normalized gene expression levels in HMGB2^+/+^ (x-axis) versus HMGB2^-/-^ (y-axis). Symbols outside the gray area indicate fold-differences larger than the 6-fold threshold. This scatter plot indicates up- and down-regulated genes in NPCs on day 1 of differentiation in the HMGB2^-/-^ compared to HMGB2^+/+^. The lower right quadrant contains a number of downregulated neurogenesis-related genes in the HMGB2^-/-^. The bar graph shows fold-change values for 10 PcG and trxG complex related genes downregulated in the HMGB2^-/-^ NSPCs at day 1 of differentiation compared to HMGB2^+/+^ cells (expression level of these genes in HMGB2^+/+^ was set at 1.


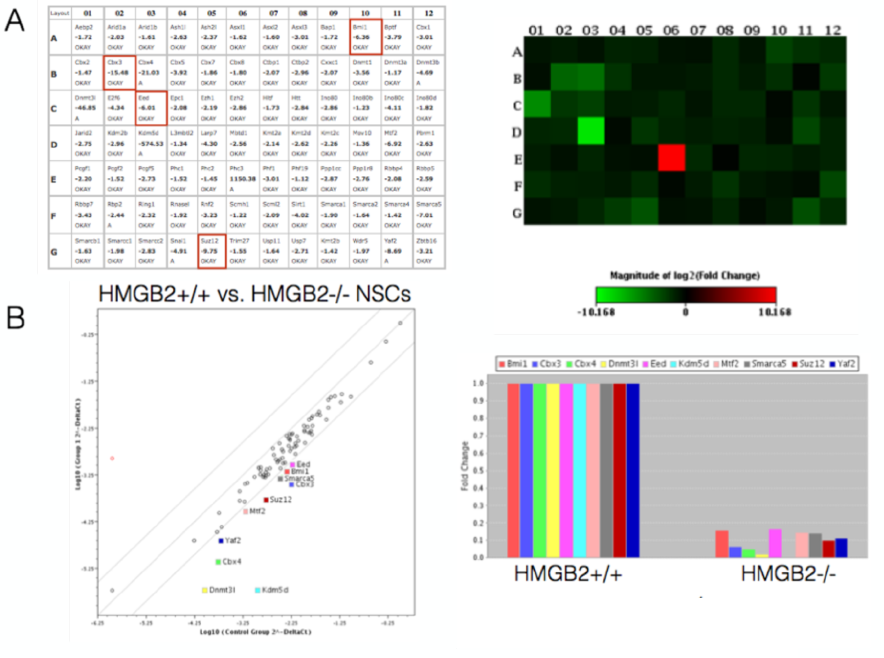


**Supplementary Figure 2: EED expression in Sox2+ neural progenitor cells (NPCs) at p1.** A) Representative images of p1 HMGB2^+/+^ (WT, n=2) and HMGB2^-/-^ (KO, n=3) at p1, with Sox2 in red, and EED in green. Scale bar is 25 um. B) Quantification of Sox2+EED+ cells over the total number of Sox2+ cells, expressed as a percentage. p= 0.283, Student's t-test.

**
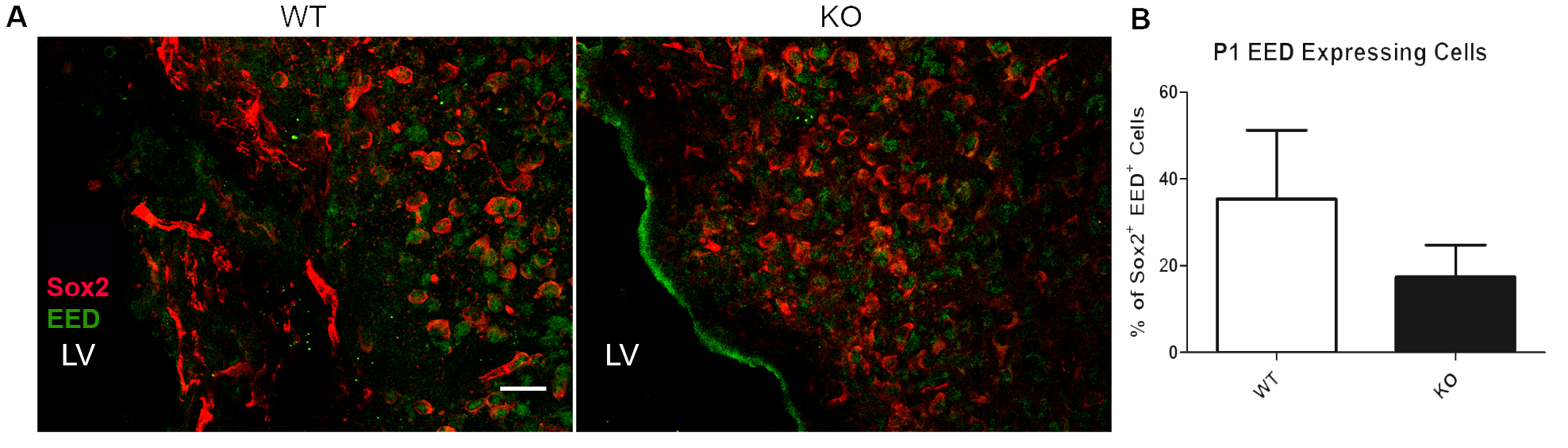
**

**Supplementary Figure 3: Histone modification H3K9me3 remains unchanged at days one and three of NSC differentiation.** Depiction of another repressive histone modification H3K9me3 **(A,B)** Western blots employing antibodies against the histone modification H3K9me3, followed by quantification of the results. Total histone H3 represents the loading control. n=2-3 biological replicates per genotype.


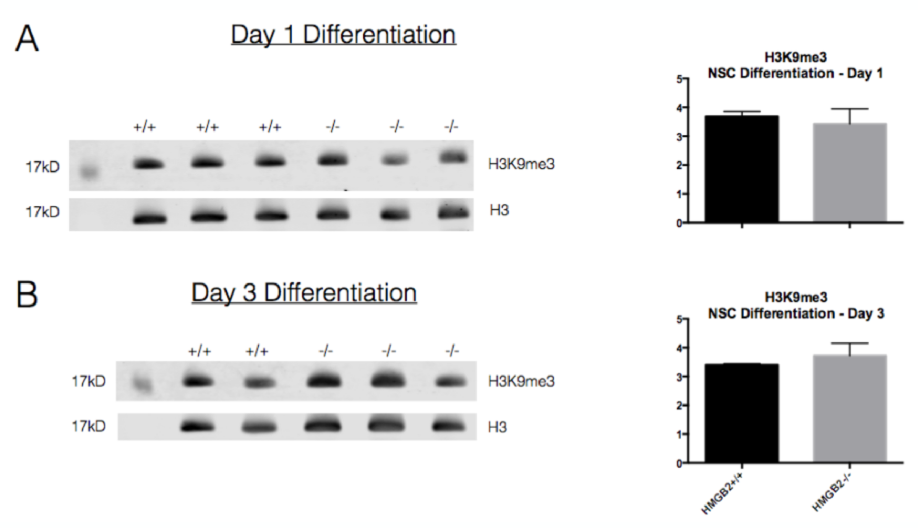


**Supplementary Figure 4: Germline ablation of HMGB2 is accompanied by ventriculomegaly.** Approximately 50% of adult HMGB2^-/-^ mice exhibit ventricular enlargement of variable penetrance as compared with 10% of WT animals (n=14).


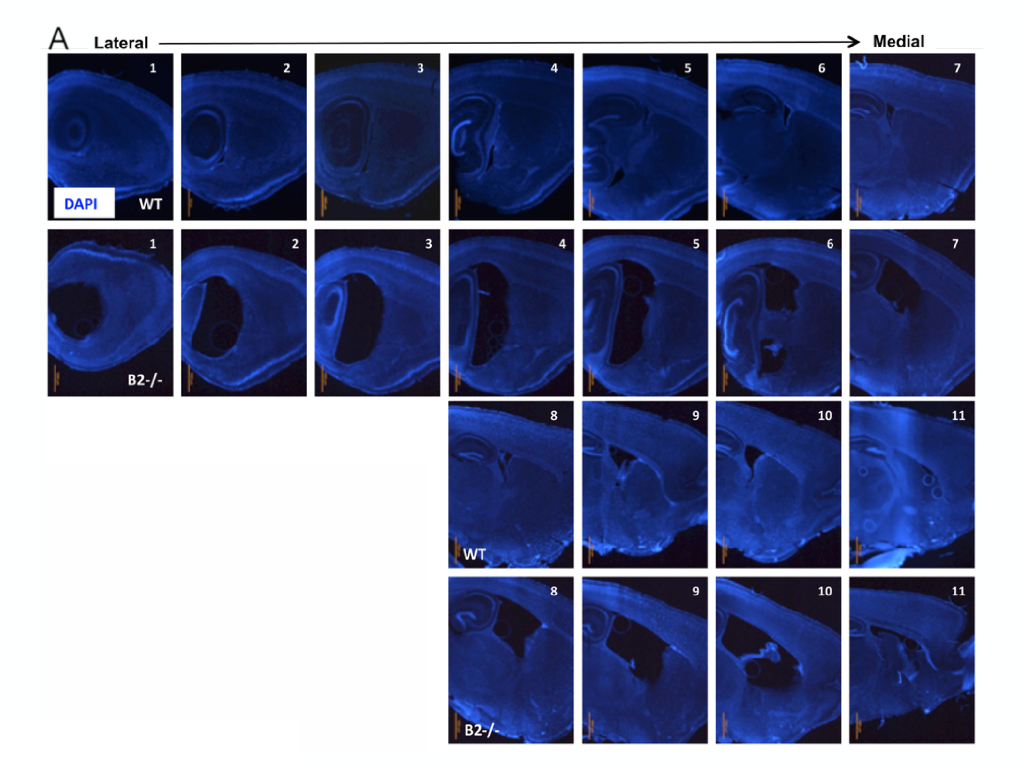


**Supplementary Figure 5: HMGB1 expression in the SVZ in HMGB2+/+ (WT) and**

**HMGB2-/- mice.** Nestin-GFP (green) control and HMGB2-/- tissues were stained for HMGB1 (red); DAPI marks nuclei (blue). Scale bar 10 um.


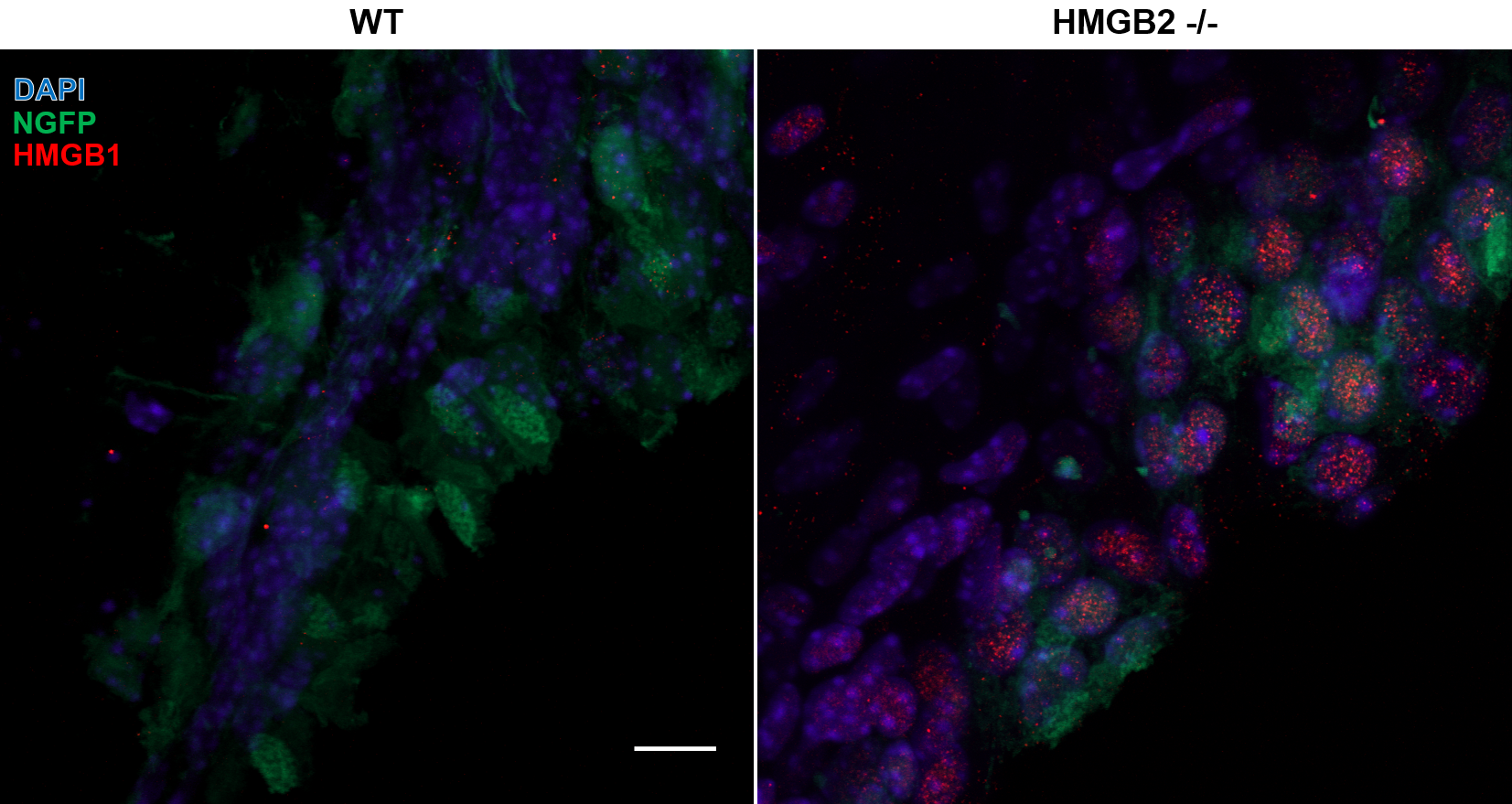


**Supplementary Table 1:** List of all primers utilized in qRT-PCR experiments.

| **Primer name** | Sequence (5’-3’) |
| --- | --- |
| BMI1_F | ATCCCCACTTAATGTGTGTCCT |
| BMI1_R | CTTGCTGTCTCCAAGTAACG |
| EED_F | ATGCTGTCAGTATTGAGAGTGGC |
| EED_R | GAGGCTGTTCACACATTTGAAAG |
| SUZ12_F | TGCCACTAGAAATTCAGAGAGCC |
| SUZ12_R | TTGTGCAGGTTTAACAGAACCA |
| CBX3_F | ACTGGACCGTCGTGTAGTGAA |
| CBX3_R | GCCCCTTGGTTTGTCAGCA |
| GAPDH_F | GCACAGTCAAGGCCGAGAAT |
| GAPDH_R | GCCTTCTCCATGGTGGTGGA |

**Supplementary Table 2:** List of antibodies.

| **Name** | **Species** | **Manufacturer** | **Working dilution** |
| --- | --- | --- | --- |
| Tuj1 | Mouse IgG1 | Cell Signaling | 1:250 |
| GFAP | Mouse IgG1 | Cell Signaling | 1:800 |
| CC1 | Mouse mAb | Abcam | 1:250 |
| NeuN | Mouse mAb | Millipore | 1:500 |
| PSA-NCAM | Mouse mAb 5A5 | DSHB Hybridoma | 1:100 |
| NG2 | Rabbit IgG | Dr. Joel Levine, SBU | 1:500 |
| GFAP | Rabbit IgG | Dako | 1:1000 |
| Aldh1l1 | Mouse mAb | Neuromab | 1:100 |
| EED | Rabbit IgG | Bioss | 1:100 |
| Sox2 | Mouse IgG1 | R&D Systems | 1:200 |
